# Increased early systemic inflammation in patients with ICU-acquired weakness

**DOI:** 10.1186/cc14552

**Published:** 2015-03-16

**Authors:** E Witteveen, L Wieske, C Verhamme, T Van der Poll, IN Van Schaik, MJ Schultz, J Horn

**Affiliations:** 1 Academic Medical Center, Amsterdam, the Netherlands

## Introduction

Inflammation may be important in the pathogenesis of ICU-acquired weakness (ICU-AW) since SIRS, sepsis and multiple organ failure are the main risk factors. Local inflammation has been found in muscle and nerve tissue of patients with ICU-AW, but little is known about the association with systemic inflammation. We hypothesized that systemic inflammation is increased in patients who develop ICUAW compared with patients who do not develop ICU-AW.

## Methods

Newly admitted ICU patients ≥48 hours on mechanical ventilation were included. Daily plasma samples were collected from leftover plasma. Muscle strength was evaluated as soon as patients were awake and attentive. ICU-AW was defined by a mean Medical Research Council score <4. IL-1β, IL-6, IL-8, IL-10, IL-13, TNFα, IFNγ, fractalkine, GM-CSF, sICAM-1, sE-selectin and sP-selectin were measured in plasma samples of days 0, 2 and 4 using cytometric bead arrays and FACS. Differences of maximum levels between patients with and without ICUAW were calculated using Mann-Whitney U tests. Principal component (PC) analysis was used to avoid multicollinearity and to reduce the set of mediators into a smaller set of PCs. To investigate whether different inflammatory profiles are associated with development of ICU-AW, we used multivariable logistic regression models of selected PCs, corrected for *a priori *selected variables, being age, gender, BMI, sepsis, SOFA score, APACHE IV score, immune insufficiency and corticosteroids.

## Results

Ninety-nine of 204 included patients developed ICU-AW. Patients with ICU-AW had higher APACHE IV and SOFA scores, a longer duration of mechanical ventilation, longer ICU stay, and died more often on the ICU compared with ICU patients without ICU-AW. Maximal levels of IL-1β, IL-6, IL-8, IL-10, TNFα, IFNγ, fractalkine and sICAM-1 were higher in patients who developed ICU-AW compared with patients who did not develop ICU-AW (univariable analysis). PC 1 to 4 derived from maximal levels explained >69% of the total variance in the data. Multivariable logistic regression models showed that PC 1 (mainly loaded by IL-6, IL-8 and IL-10) and PC 4 (mainly loaded by sP-selectin) were significantly higher in patients with ICU-AW compared with patients without ICU-AW (OR of 1.27 (95% CI = 1.02 to 1.60) and 1.55 (1.06 to 2.27) respectively).

## Conclusion

Development of ICU-AW is associated with increased systemic inflammation in the first days after ICU admission.

## Acknowledgement

This research was supported by the Center for Translational Molecular Medicine (MARS).

